# Supplementary material for: Longitudinal trajectories of depressive symptoms in children are associated with baseline inflammation and HIV status
Source: Commun Health. 2026 Jul 3;1(1):9. doi: 10.1038/s44528-026-00008-3 (PMC13347800; doi:10.1038/s44528-026-00008-3)
Supplement: Supplementary file 1 — Supplementary Materials [file 44528_2026_8_MOESM1_ESM.pdf]

Supplementary Materials

Article title: Longitudinal trajectories of depressive symptoms in children are associated with baseline inflammation and HIV status

Authors: Arish Mudra Rakshasa-Loots, Sarah K. Zalwango, Simon R. Cox, Alla Sikorskii, Bruno Giordani, Jorem E. Awadu, and Amara E. Ezeamama

Table of contents

Item	Page
Supp Figure 1: Outcome distributions before transformation.	1
Supp Figure 2: Outcome distributions after log transformation.	2
Supp Figure 3: Visualisation of PHQ-9 trajectories with clinical thresholds for high and low CRP categorisation.	3
Supp Table 1: Full results from latent growth curve models.	4
Supp Table 2: Proportion of missingness in PHQ-9 scores over time.	5
Supp Table 3: Full results from latent growth curve models including only participants with at least three PHQ-9 scores available.	6

Supplementary Figure 1

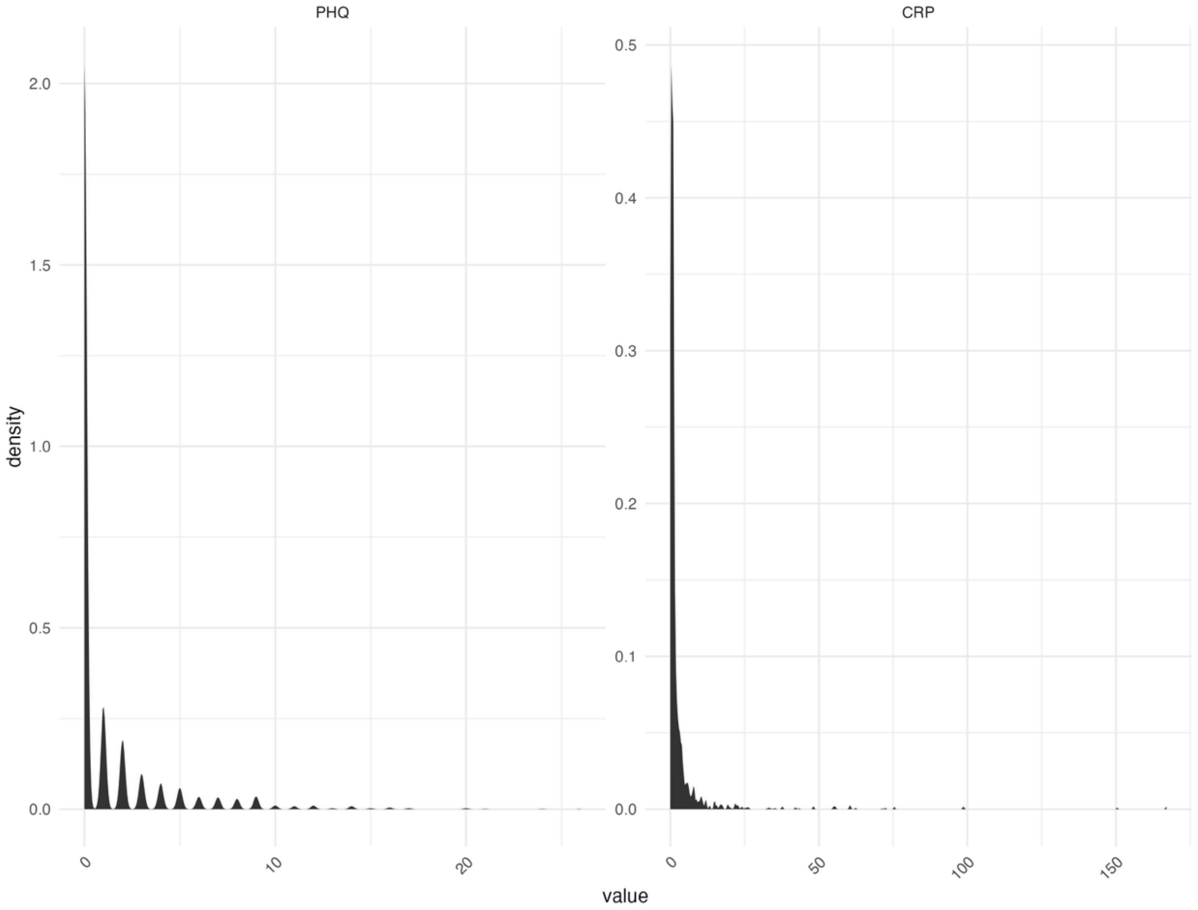

Supp Figure 1: Outcome distributions before transformation. Distribution densities are shown for PHQ-9 scores (left) and CRP concentrations (right).

Supplementary Figure 2

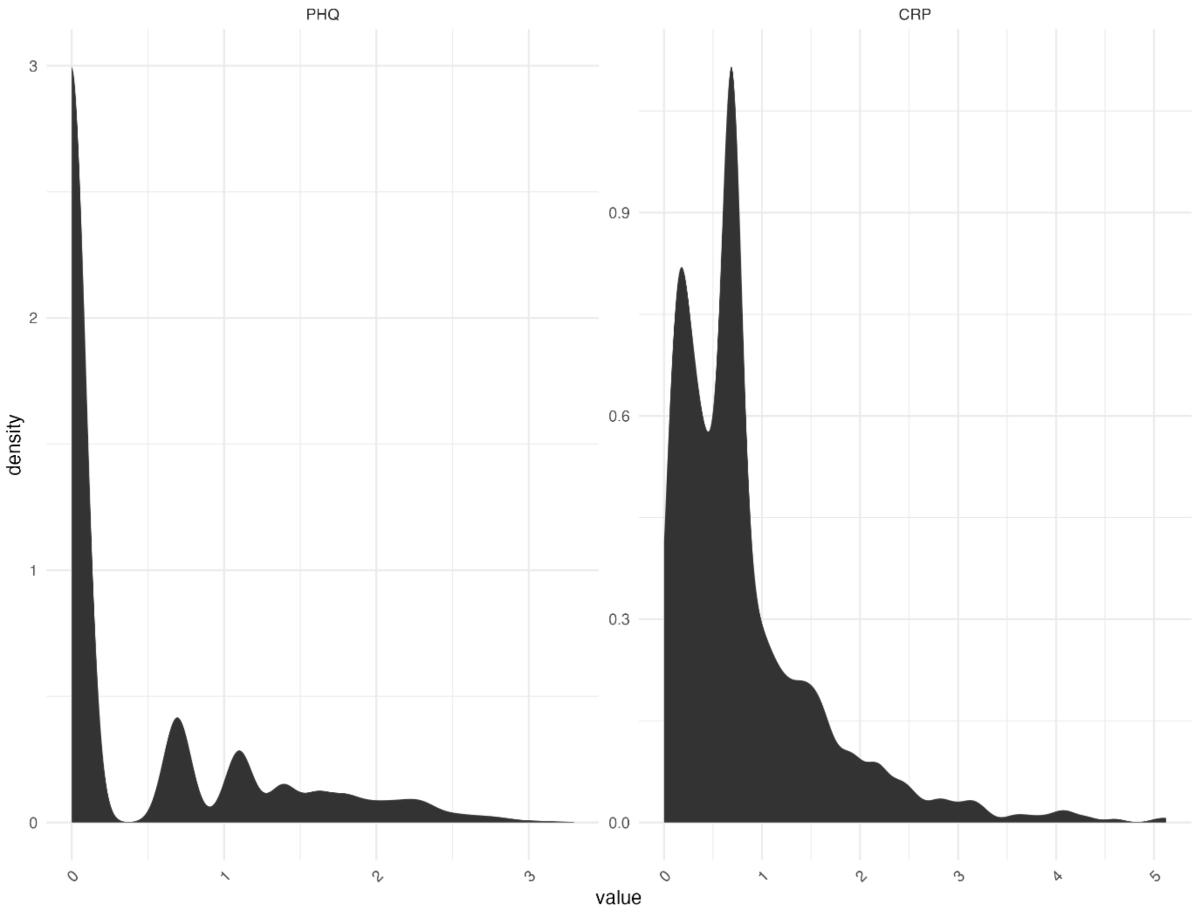

Supp Figure 2: Outcome distributions after log transformation. Distribution densities are shown for PHQ-9 scores (left) and CRP concentrations (right).

Supplementary Figure 3

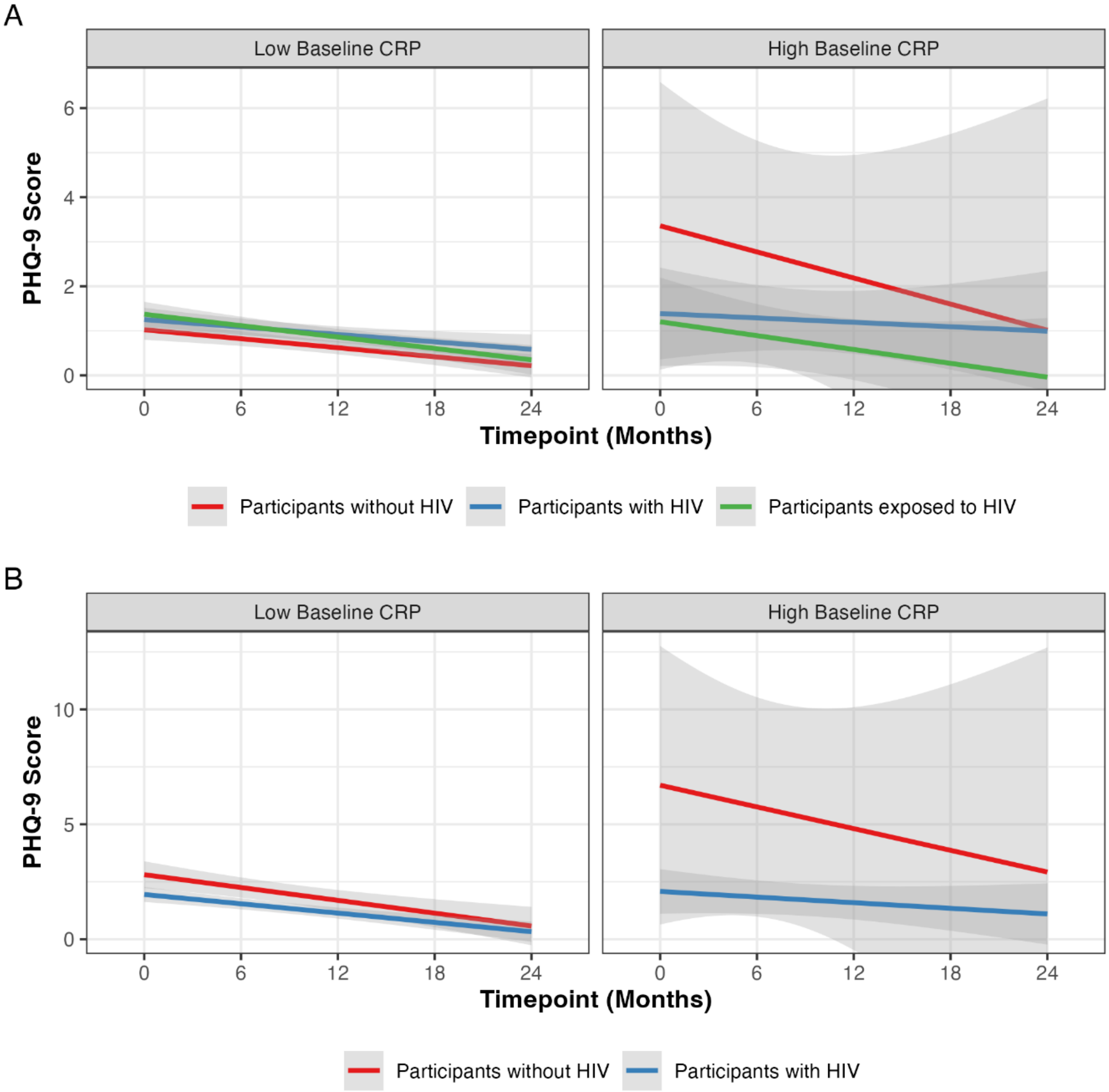

Supp Figure 3: Mean longitudinal trajectories of depressive symptoms as functions of HIV status and clinical thresholds of baseline CRP concentrations among (A) children and (B) adults. Participants with baseline CRP concentrations > 10 mg/L were categorised as “High Baseline CRP” and those with CRP concentrations ≤ 3 mg/L were categorised as having “Low Baseline CRP”. Individual lines represent the fitted linear models with shaded grey areas representing 95% confidence intervals.

Supplementary Table 1

Full model outputs. Results from latent growth curve modelling of PHQ-9 score trajectories in children and adult caregivers. *P* values were not corrected for multiple comparisons.

CHILDREN								
**LHS**	**op**	**RHS**	**est**	**se**	**z**	**pvalue**	**ci.lower**	**ci.upper**
Intercept	~	CRP	0.069	0.047	1.465	0.143	-0.023	0.161
Intercept	~	HIV.status	0.048	0.045	1.085	0.278	-0.039	0.136
Intercept	~	CRP:HIV.status	-0.094	0.044	-2.123	0.034	-0.181	-0.007
Intercept	~	age	0.151	0.045	3.315	0.001	0.062	0.24
Intercept	~	sex	-0.04	0.044	-0.906	0.365	-0.127	0.047
Slope	~	CRP	-0.069	0.034	-1.996	0.046	-0.136	-0.001
Slope	~	HIV.status	0.033	0.031	1.051	0.293	-0.029	0.095
Slope	~	CRP:HIV.status	0.091	0.031	2.914	0.004	0.03	0.152
Slope	~	age	-0.121	0.033	-3.681	0	-0.186	-0.057
Slope	~	sex	-0.009	0.031	-0.292	0.77	-0.07	0.052
Intercept	~~	Intercept	0.841	0.087	9.684	0	0.671	1.011
Slope	~~	Slope	0.224	0.039	5.679	0	0.146	0.301
Intercept	~~	Slope	-0.272	0.051	-5.37	0	-0.371	-0.173
Intercept	~1		0.178	0.045	3.933	0	0.089	0.266
Slope	~1		-0.172	0.032	-5.358	0	-0.235	-0.109
ADULTS								
**LHR**	**op**	**RHS**	**est**	**se**	**z**	**pvalue**	**ci.lower**	**ci.upper**
Intercept	~	CRP	-0.048	0.053	-0.901	0.367	-0.152	0.056
Intercept	~	HIV.status	-0.075	0.053	-1.409	0.159	-0.179	0.029
Intercept	~	CRP:HIV.status	0.068	0.056	1.215	0.224	-0.042	0.178
Intercept	~	age	-0.013	0.056	-0.227	0.82	-0.122	0.096
Intercept	~	sex	-0.093	0.056	-1.68	0.093	-0.202	0.016
Slope	~	CRP	0.018	0.034	0.526	0.599	-0.048	0.084
Slope	~	HIV.status	0.042	0.032	1.286	0.198	-0.022	0.105
Slope	~	CRP:HIV.status	0.041	0.037	1.126	0.26	-0.031	0.113
Slope	~	age	0.012	0.032	0.382	0.702	-0.051	0.076
Slope	~	sex	0.019	0.032	0.595	0.552	-0.044	0.081
Intercept	~~	Intercept	1.009	0.101	10.02	0	0.812	1.206
Slope	~~	Slope	0.186	0.034	5.491	0	0.119	0.252
Intercept	~~	Slope	-0.376	0.053	-7.157	0	-0.479	-0.273
Intercept	~1		0.246	0.054	4.584	0	0.141	0.352
Slope	~1		-0.306	0.032	-9.552	0	-0.369	-0.243

Supplementary Table 2

Missingness in PHQ-9 scores. Proportion and pattern of missingness in PHQ-9 scores over time.

CHILDREN		
**Missing timepoints**	***N***	**% of total sample**
0	1	0.1
1	83	9.6
2	131	15.2
3	111	12.9
4	536	62.2
ADULTS		
**Missing timepoints**	***N***	**% of total sample**
0	18	4.1
1	146	33.3
2	42	9.6
3	13	3.0
4	220	50.1

Supplementary Table 3

Complete case analysis results. Results from latent growth curve modelling of PHQ-9 score trajectories in children and adult caregivers when only including cases with complete data on PHQ-9 scores over time. *P* values were not corrected for multiple comparisons.

CHILDREN								
**LHS**	**op**	**RHS**	**est**	**se**	**z**	**pvalue**	**ci.lower**	**ci.upper**
Intercept	~	CRP	0.193	0.088	2.198	0.028	0.021	0.365
Intercept	~	HIV.status	0.09	0.084	1.077	0.281	-0.074	0.255
Intercept	~	CRP:HIV.status	-0.232	0.085	-2.723	0.006	-0.398	-0.065
Intercept	~	age	0.365	0.106	3.449	0.001	0.158	0.573
Intercept	~	sex	-0.086	0.084	-1.024	0.306	-0.252	0.079
Slope	~	CRP	-0.109	0.047	-2.315	0.021	-0.201	-0.017
Slope	~	HIV.status	-0.002	0.045	-0.039	0.969	-0.091	0.087
Slope	~	CRP:HIV.status	0.116	0.045	2.595	0.009	0.028	0.204
Slope	~	age	-0.139	0.058	-2.398	0.016	-0.252	-0.025
Slope	~	sex	0.029	0.045	0.647	0.518	-0.06	0.118
Intercept	~~	Intercept	1.006	0.143	7.023	0	0.725	1.287
Slope	~~	Slope	0.226	0.047	4.837	0	0.135	0.318
Intercept	~~	Slope	-0.365	0.072	-5.089	0	-0.505	-0.224
Intercept	~1		0.053	0.093	0.569	0.57	-0.13	0.236
Slope	~1		-0.156	0.051	-3.066	0.002	-0.256	-0.056
ADULTS								
**LHR**	**op**	**RHS**	**est**	**se**	**z**	**pvalue**	**ci.lower**	**ci.upper**
Intercept	~	CRP	-0.028	0.084	-0.329	0.742	-0.193	0.138
Intercept	~	HIV.status	-0.122	0.08	-1.524	0.127	-0.278	0.035
Intercept	~	CRP:HIV.status	0.033	0.093	0.358	0.721	-0.15	0.217
Intercept	~	age	0.023	0.078	0.295	0.768	-0.13	0.176
Intercept	~	sex	-0.06	0.075	-0.796	0.426	-0.207	0.088
Slope	~	CRP	0.004	0.043	0.092	0.926	-0.081	0.089
Slope	~	HIV.status	0.052	0.041	1.285	0.199	-0.028	0.132
Slope	~	CRP:HIV.status	0.055	0.048	1.138	0.255	-0.039	0.148
Slope	~	age	0.001	0.039	0.025	0.98	-0.076	0.078
Slope	~	sex	0.008	0.038	0.205	0.838	-0.067	0.082
Intercept	~~	Intercept	0.988	0.125	7.899	0	0.743	1.234
Slope	~~	Slope	0.18	0.035	5.086	0	0.111	0.249
Intercept	~~	Slope	-0.364	0.06	-6.089	0	-0.482	-0.247
Intercept	~1		0.176	0.077	2.284	0.022	0.025	0.327
Slope	~1		-0.272	0.04	-6.876	0	-0.35	-0.195
